# Identification of High-Affinity Inhibitors of Cyclin-Dependent Kinase 2 Towards Anticancer Therapy

**DOI:** 10.3390/molecules24244589

**Published:** 2019-12-15

**Authors:** Taj Mohammad, Sagar Batra, Rashmi Dahiya, Mohammad Hassan Baig, Irfan Ahmad Rather, Jae-June Dong, Imtaiyaz Hassan

**Affiliations:** 1 Centre for Interdisciplinary Research in Basic Sciences, Jamia Millia Islamia, New Delhi 110025, India; taj144796@st.jmi.ac.in (T.M.); rashmidahiya285@gmail.com (R.D.); 2 Amity Institute of Biotechnology, Amity University Rajasthan, Rajasthan 303002, India; sagar.sb885@gmail.com; 3 Department of Family Medicine, Yonsei University College of Medicine, Gangnam Severance Hospital, 211 Eonju-Ro, Gangnam-Gu, Seoul 06273, Korea; mohdhassanbaig@gmail.com (M.H.B.); s82tonight@naver.com (J.-J.D.); 4 Department of Biological Sciences, Faculty of Science, King Abdulaziz University, P.O. Box 80141, Jeddah 21589, Saudi Arabia; erfaan21@gmail.com

**Keywords:** cyclin-dependent kinase 2, molecular docking, drug-likeness, drug design and discovery, molecular dynamics simulation, kinase inhibitor, cancer

## Abstract

Cyclin-dependent kinase 2 (CDK2) is an essential protein kinase involved in the cell cycle regulation. The abnormal activity of CDK2 is associated with cancer progression and metastasis. Here, we have performed structure-based virtual screening of the PubChem database to identify potent CDK2 inhibitors. First, we retrieved all compounds from the PubChem database having at least 90% structural similarity with the known CDK2 inhibitors. The selected compounds were subjected to structure-based molecular docking studies to investigate their pattern of interaction and estimate their binding affinities with CDK2. Selected compounds were further filtered out based on their physicochemical and ADMET properties. Detailed interaction analysis revealed that selected compounds interact with the functionally important residues of the active site pocket of CDK2. All-atom molecular dynamics simulation was performed to evaluate conformational changes, stability and the interaction mechanism of CDK2 in-complex with the selected compound. We found that binding of 6-*N*,6-*N*-dimethyl-9-(2-phenylethyl)purine-2,6-diamine stabilizes the structure of CDK2 and causes minimal conformational change. Finally, we suggest that the compound (PubChem ID 101874157) would be a promising scaffold to be further exploited as a potential inhibitor of CDK2 for therapeutic management of cancer after required validation.

## 1. Introduction

Protein kinases play an important role in catalyzing the phosphorylation of many substrate proteins which in turn regulate biological processes [1]. In general, phosphorylation takes place on the specific amino acid residue, namely tyrosine, serine and threonine, which generally control the cell cycle and cell development. Cyclin-dependent kinase 2 (CDK2) is one such protein kinase that is known to control the cell cycle by initiating the late G_1_ phase to enter the G_1_-S transition phase [2]. Governance of the cell cycle to G_2_ from the S phase, or replicating phase, is promoted by cyclin A [3]. During DNA replication, spindle fibers are formed by the help of centrioles and procentrioles mainly depending on the proper functioning of CDK2 [4]. Thus, the association of CDK2 with cyclin E and A specifically controls the regulation of the G_1_-S phase of the cell cycle.

The role of CDK2 in cell division is especially important as it prevents the dysfunction of cell cycle. The overexpression of CDK2 causes abnormal regulation of the cell cycle, which is directly associated with hyperproliferation of cancer cells. There are many evidences supporting the idea of targeting CDK2 to control cancer progression [5]. The dysregulation of CDK2 is observed in the breast and other cancer, thereby making it a potential drug target for anticancer therapy [6,7].

The cellular machinery works in a precise replication and transcription process which regulates cell division [8]. Cyclin E and A are synthesized by the E2F transcriptional factor, which is a cell division promoting factor [9]. Thus, the overexpression of E2F results in the excessive release of cyclins [10]. The higher amount of processed cyclin E and A forms the complex with CDK2 which causes malignancies. Complexing with cyclin E is a root cause of high-level expression of CDK2 in colorectal carcinoma [7,11]. Retinoblastoma protein, a tumor suppressor known for its capability of binding with E2F to inhibit its function, was found to be phosphorylated by CDK2/cyclin E complex, causing the cell cycle progression of an under-developed cell and tumor in the respective bound areas [12].

Structurally, CDK2 consists of two lobes, the N-terminal lobe (Met1-Phe80), composed of five antiparallel β-sheets and a small PSTAIRE helix, and the C-terminal lobe which is dominated by α-helices where the active site cavity lies [13]. The ATP-binding site in CDK2 is highly conserved and regulated by Lys33 and Asp145. The glycine-rich motif, containing Thr14 and Tyr15 is in high proximity to the γ-phosphate of the ATP, promoting phosphorylation. The substrate-binding site of CDK2 faces a residual blockage because of the presence of a T-loop (Gly152-Ala170) [13]. This T-loop contains many critical residues inside a narrow cavity near the active site where it forms a central network of hydrogen bonds with CDK2 and other cyclins. Thr160 present on the top of the T-loop is a key residue for phosphorylation and activation of the receptor. The loop being flattened prepares a hindrance for the substrate to bind to (a self-inhibitory nature), thereby binding of cyclin A causes considerable conformational change to expose the phosphorylating group to the solvent area and activating the complex [14,15]. The ‘PSTAIRE’ motif, a glycine-rich region near the ATP site and C-terminal helix connecting the T-loop, is a defined conserved region that regulates the binding of ligands to the protein.

In recent years, a number of CDK2 inhibitors have been developed and are under clinical evaluation [16]. Recently, several strategies have been integrated to develop different classes of inhibitors for CDK2 to control glioma and colorectal cancers [17]. Still, the available inhibitors of CDK2 are not selective and possess on- and off-target effects. Hence, there is a need to develop safe and selective inhibitors of CDK2 for the therapeutic management of cancer and associated disorders.

Here, we utilized a structure-based virtual screening approach to identify potential inhibitors of CDK2. We performed all-atom molecular dynamics (MD) simulation to investigate the dynamics of CDK2 and the binding prototype of the selected compound, to evaluate the structural stability of the docked complex [18]. Our strategy of structure-based virtual screening of the PubChem database may open new avenues in the identification of safe and effective inhibitors of CDK2 for the development of therapeutic molecules for cancer treatment. The identified compound with improved pharmacological features can be further evaluated for the development of selective CDK2 inhibitors with required modifications.

## 2. Results and Discussion

### 2.1. Interaction Analysis of CDK2-Ligand Crystal Structures

A total of 61 PDB entries of CDK2 were retrieved for interaction analyses of co-crystallized ligands (Table S1). We noticed that Leu83 appeared in 54 entries of co-crystallized CDK2. Leu83 is a known site for nucleotide-binding, along with Ile 10, where many co-crystallized ligands are interacting. Lys33 and Asp86 are found in the ATP-binding site of CDK2 [19,20]. We selected the CDK2 structure with PDB 2R3I for docking and simulation studies because of its non-mutated monomeric form with a higher resolution (1.28 Å). This structure is co-crystallized with ligand 5-(2-fluorophenyl)-*N*-(pyridin-4-ylmethyl)pyrazolo [1,5-a]pyrimidin-7-amine along with 3-sulfinoalanine and an acetyl group.

### 2.2. Screening of CDK2 Inhibitor-Like Compounds

Some of the known inhibitors of CDK2 viz., Olomoucine (a plant derivative purine analog) [21], Hymenialdisine [22], SU9516 [23], and Bosutinib [24] were selected to screen the PubChem database. The chemical structures of these CDK2 inhibitors and their biological activity are shown in Table S5. CDK2 inhibitor-like compounds with structural similarity of more than 90% were identified from the PubChem database, where a total of 2117 compounds were retrieved. Docking of these compounds with CDK2 predicts a preferred orientation at binding pocket. Here we found many (~50) compounds showing appreciable binding affinities (≥−8.6 kcal/mol) and preferentially occupying the substrate-binding site of CDK2. These compounds were subjected to further assessment and interaction analysis (Table S2).

### 2.3. Assessment and Interaction Analysis

We assessed the physicochemical properties of the above-selected compounds. At this stage, 48 compounds were following the Lipinski’s rule of five for their drug-likeliness (Table S3). These compounds were subjected to ADMET (Absorption, distribution, metabolism, excretion and toxicity) analysis where only four compounds showed admirable ADMET properties. Selected compounds are predicted to be non-carcinogenic (Table 1 and Table S4).

**Figure 1:**
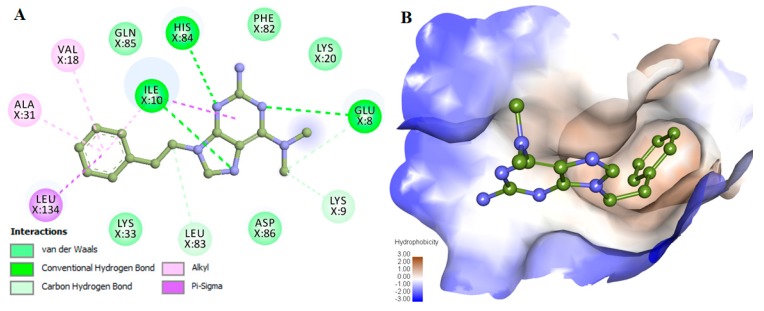
Interactions of compound 101874157 with cyclin-dependent kinase 2 (CDK2). (**A**) 2D representation of CDK2 interacting residues, and (**B**) Surface view of CDK2 in-complex with 101874157. The structures were drawn in Discovery Studio by using the docked coordinates of the CDK2-101874157 complex.

**Figure 2:**
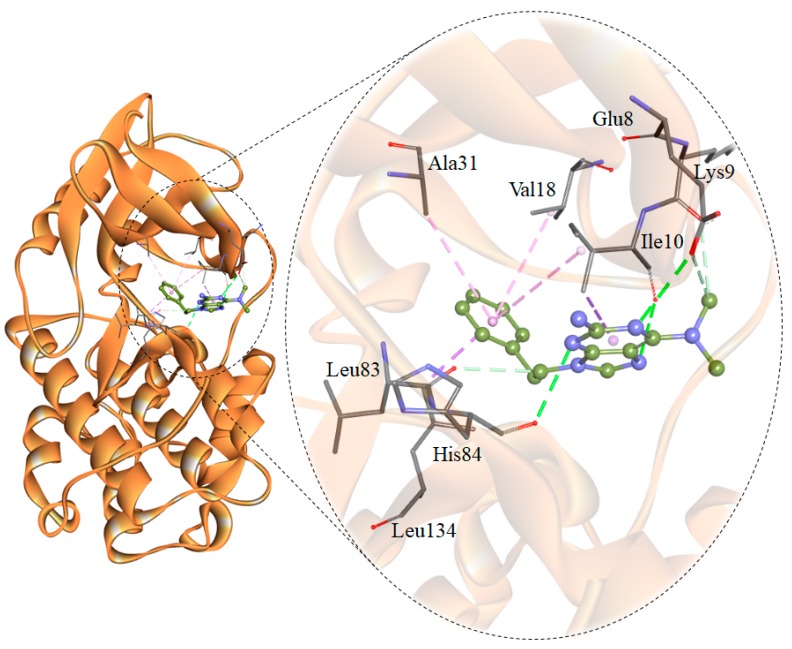
Structural representation of CDK2 in-complex with 101874157. The structure was drawn in Discovery Studio by using the docked coordinates of the CDK2-101874157 complex.

The interaction analysis revealed some compounds showing binding orientation like co-crystallized inhibitors of CDK2. Based on the interaction analysis, we identified a compound, 6-*N*,6-*N*-dimethyl-9-(2-phenylethyl)purine-2,6-diamine (PubChem ID 101874157) having all requisites for a drug molecule. The compound 101874157 has a total of 11 spatial pharmacophore features as it contains 3 aromatics, 3 hydrophobics, 1 donor, 3 acceptors, and 1 positive group. This compound is most suitable after comparing all the possible arrangements and has the capability for efficient binding with the CDK2 binding-pocket. This is particularly due to forming hydrogen bonds with Leu83 as well as the other functional residues with the most preferential conformation. It forms six hydrogen bonds (three conventional and three carbon-hydrogen) with Leu83, His84, Lys9, Ile10, Glu8, and other non-covalent π-Alkyl, π-sigma and van der Waals interactions with Val18, Ala31, Leu134, Ile10, Lys33, Asp86, Phe82, Lys20 and Gln85 of CDK2 (Figure 1 and Figure 2**)**. A comparative analysis of the interaction of the selected compound and known inhibitors with CDK2 is illustrated in Figure S1. The study reveals a common set of interactions of compound 101874157 and known CDK2 inhibitors. The interaction between these residues may decrease the catalytic activity of CDK2 [25]. The compound occupies the internal cavity and binds with CDK2 with an appreciable affinity (Figure 1B). Figure S2 shows the ligand behavior in the binding pocket of CDK2 and the dynamics of conformation before and after MD simulation. We observed that the ligand stays in the CDK2 binding pocket without switching throughout the simulation (Figure S2). 

### 2.4. The Potential Energy of the Systems

The average potential energy of CDK2 and the CDK2-101874157 complex was estimated to determine the equilibration and stability of both systems. An average of the potential energy for CDK2 and CDK2-101874157 complex was found to be −863,562 kJ/mol and −844,551 kJ/mol, respectively. Systematic and energetic parameters, volume, density and enthalpy were also calculated after the simulation (Table 2).

### 2.5. Structural Deviation and Compactness

**Figure 3:**
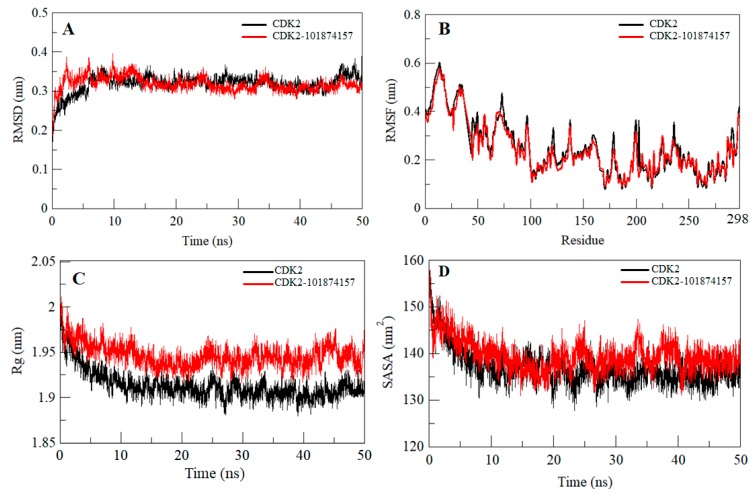
Dynamics of CDK2 structure after binding of the compound 101874157. (**A**) Time evaluation of root-mean-square deviation (RMSD) of CDK2 and CDK2-101874157 complex. (**B**) Root-mean-square fluctuation (RMSF) of CDK2 before and after compound binding. (**C**) Time evolution of the radius of gyration. (**D**) Solvent accessible surface area (SASA) plot of CDK2 before and after compound binding. Values were obtained from the 50 ns molecular dynamics (MD) simulation time scale. Black and red represent free CDK2 and CDK2-101874157 complex, respectively.

Any small molecule can induce a large conformational change in a protein after binding [26,27,28,29]. Root-mean-square deviation (RMSD) is an important approach to estimate the structural deviation and stability of a protein structure [30,31,32,33,34,35]. The average RMSD for CDK2 and CDK2-101874157 complex was found to be 0.32 nm and 0.31 nm, respectively (Table 2). The RMSD plot suggests that the binding of the selected compound stabilizes the CDK2 structure and leads to a few conformational changes (Figure 3A). A little fluctuation can be seen up to 15 ns in RMSD upon compound binding which is possibly due to its initial orientation of the compound in the CDK2 binding pocket.

To investigate the local vibrations in CDK2 before and after binding of compound 101874157, an average of the residual fluctuations in CDK2 was calculated and plotted as the root-mean-square fluctuation (RMSF) (Figure 3B). The RMSF plot showed several residual fluctuations in different regions of CDK2. We found a decrease in RMSF of CDK2 upon compound binding throughout the simulation at region spanning from N- terminal to C-terminal.

The radius of gyration (*R_g_*) is a parameter that is directly associated with the overall conformational shape of a protein and used to get insights into protein stability and folding behavior [36,37]. We assessed the stability of CDK2 and CDK2-101874157 complex by computing their *R_g_*. The averages of the *R_g_* values for free CDK2 and CDK2-101874157 complex were found to be 1.91 nm and 1.94 nm, respectively. The *R_g_* plot suggested a little change in the packing of CDK2 in-presence of the compound. The complex shows a slightly higher *R_g_* compared to free CDK2 with stable equilibrium throughout the simulation (Figure 3C). Here, no conformational shift was observed in the *R_g_* plot which suggests an insignificant structural deviation in CDK2 upon compound binding.

Solvent accessible surface area (SASA) of a protein is the area that directly interacts with its surrounding solvent [38,39]. The SASA of a protein is directly interrelated to its *R_g_*. An average of the SASA values for CDK2 and CDK2-compound complexes was calculated during the 50 ns MD simulation. The average SASA for free CDK2 and CDK2-101874157 complex was found to be 136.81 nm^2^ and 139.49 nm^2^, respectively. A small increase in SASA was observed because of the exposure of some of the internal residues due to conformational change in the protein after binding with compound 101874157 (Figure 3D).

### 2.6. Hydrogen Bonds Analysis

**Figure 4:**
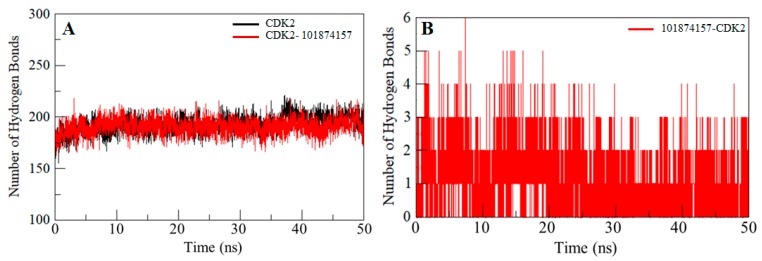
Time evolution and stability of hydrogen bonds formed within 3.5 Å. (**A**) Intramolecular hydrogen bonds in CDK2, and (**B**) hydrogen bonds between compound 101874157 and CDK2.

Intramolecular hydrogen bonds in a protein are required for stability and overall conformation [40,41,42]. Hydrogen bonding is utilized to get insight into the protein-ligand interaction mechanism with special attention to the specificity of the interaction. To validate the stability of CDK2 and the CDK2-101874157 complex, hydrogen bonds paired within 0.35 nm during the simulation were calculated and plotted. An average number of intramolecular hydrogen bonds in the CDK2 before and after compound binding were found to be 193 and 191, respectively, whereas two hydrogen bonds are present between the compound 101874157 and CDK2 throughout the simulation. However, compound 101874157 forms 4–5 hydrogen bonds to the active pocket residues of CDK2 with higher fluctuation, and 2–3 hydrogen bonds with the least fluctuation. This study also supports our molecular docking results (Figure 4).

### 2.7. Evaluation of Secondary Structures

**Figure 5:**
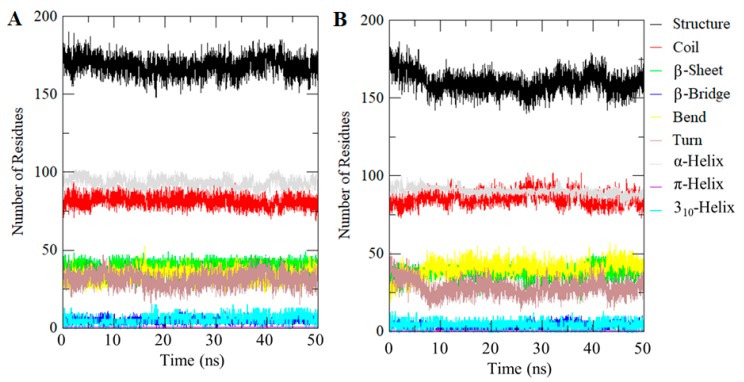
Secondary structure content of (**A**) Free CDK2, and (**B**) CDK2-101874157 complex. Structure = α-helix + β-sheet + β-bridge + Turn.

Investigating the dynamics of the secondary structure content of a protein can be carried out to understand its conformational behavior and folding mechanism. We calculated the secondary structural changes in the CDK2 upon binding of compound 101874157. The structural components in free CDK2 remain almost constant and equilibrated throughout the simulation of 50 ns (Figure 5). However, a small decrease can be seen in the α-helix and β-sheets content of CDK2 upon compound binding (Figure 5B). The average number of residues participate in secondary structure formation in the case of CDK2-101874157 complex were decreased slightly as compared to free CDK2 (Figure 5; Table 3). However, no major change was seen in the secondary structure of CDK2 upon binding of compound 101874157 which shows strong stability of CDK2-101874157 complex. Taken together, the specific pharmacological action of the selected compound 101874157 is yet unknown but the core pharmacophores we represented here could potentially be used to develop CDK2 inhibitors [16,43,44]. Hence, we assume that the development of selective inhibitors of CDK2 using such a strategy of structure-based drug design may open a newer avenue for cancer therapy.

## 3. Materials and Methods

### 3.1. Materials

Bioinformatics software, such as MGL Tools, Discovery Studio, VMD, Swiss-PDB Viewer, and QtGrace, were used in retrieval, evaluation and analysis of the data. The atomic structure of CDK2 was downloaded from the Protein Data Bank (PDB ID: 2R3I) and preprocessed in PyMod 2.0 to reconstruct the structure. Three-dimensional structures of compounds were taken from the PubChem database in the processed form [45]. The pharmacophore features of the selected compound were generated through the PharmaGist Webserver (https://bioinfo3d.cs.tau.ac.il/PharmaGist/). The AutoDock Vina tool [46] was used for molecular docking. All-atom MD simulations were performed on CDK2 alone and with one of the selected compounds at 300 K at the molecular mechanics level using GROMOS96 43a1 force-field in GROMACS 5.1.2 for 50 ns of time.

### 3.2. Interaction Analysis of Co-Crystallized Ligands

To identify the key residues of CDK2 participating in ligand binding, all the co-crystallized structures available in the PDB were retrieved and their binding patterns were analyzed in detail using the Protein Structure Analysis Package (PSAP)-web-based suite [47]. In many structures, multiple ligands were found to interact with CDK2. Here, we identified some functionally important residues of CDK2 which dominantly participate to bind with the known inhibitors.

### 3.3. Retrieval of CDK2 Inhibitor-Like Compounds

We retrieved some of the known inhibitors of CDK2 from the DrugBank to identify the PubChem compounds having a close relation to them. The known inhibitors of CDK2 viz., Olomoucine (2-(2-hydroxyethylamino)-6-benzylamino-9-methylpurine) [21], Hymenialdisine (Pyrrolo(2,3-c)azepin-8(1H)-one,4-(2-amino-1,5-dihydro-5-oxo-4H-imidazol-4-ylidene)-2-bromo-4,5,6,7-tetrahydro-) [22], SU9516 (3-[1-(3H-Imidazol-4-yl)-meth-(*Z*)-ylidene]-5-methoxy-1,3-dihydro-indol-2-one) [23], and Bosutinib (4-((2,4-dichloro-5-methoxyphenyl)amino)-6-methoxy-7-(3-(4-methyl-1-piperazinyl)propoxy)-3-quinolinecarbonitrile) [24] were selected to screen the PubChem database. The selection criteria for the known CDK2 inhibitors were structural similarity and their inhibitory potential. We chose only those compounds which are reported to inhibit CDK2 significantly and are somehow similar in their chemical structure. We retrieved all the compounds from the PubChem database having more than 90% structural similarity with the above selected known inhibitors of CDK2. Finally, we selected those compounds for further screening which were structurally close (having at least 90% structural similarity) to the known inhibitors of CDK2.

### 3.4. Virtual Screening Using Molecular Docking Approach

Molecular-docking-based virtual screening was performed to identify suitable compounds that strongly bind to CDK2. Subsequently, their bound conformations and binding affinities were estimated. The structure preparation of the receptor was initiated with the removal of co-crystallized ligands and water molecules before energy minimization using the MGL tools and Swiss PDB Viewer [48]. Hydrogen atoms were added to the polar groups of the protein followed by adding the Kollman charges. Docking was structurally blind in AutoDock Vina with a grid of size 48, 40 and 60 Å, centralized at −1.05, 30.06 and 19.28 for X, Y, and Z coordinates, respectively. The grid spacing was set to 1.00 Å with the exhaustiveness of 8 where all the compounds were free to move and search for their favorable binding site(s) in CDK2 [49].

### 3.5. Hit-Selection and Drug-Ability Assessment

The compounds obtained from docking were analyzed on the basis of their physicochemical and ADMET properties using the SwissADME [50] and pkCSM [51] webservers. Non-carcinogenicity of the compounds was predicted through the CarcinoPred-EL [52]. Interaction analysis of the compounds was carried out using Discovery Studio followed by splitting all possible conformers using Vina Split application to analyze their binding pattern with CDK2 to get selective compounds specifically interacting with the important residues [53].

### 3.6. MD Simulations

We performed all-atom MD simulations on free CDK2 and the CDK2-101874157 complex for 50 ns to evaluate stability and conformational changes in CDK2 along with their interaction mechanisms under explicit solvent conditions. The topology parameters for compound 101874157 were generated through the PRODRG server and merged into the protein topology to make the CDK2-101874157 complex system. Both systems were soaked in a cubic box with the Simple Point Charge (spc216) water model to simulate aqueous surroundings. Energy minimization for 1500 steps of steepest descent was performed on both systems before equilibration at 300 K. The temperature of both systems was subsequently raised from 0 to 300 K during the equilibration period of 100 ps at constant volume under periodic boundary conditions with a stable environment of 1 bar pressure. Final MD run was performed for 50,000 ps for both systems and the resulting trajectory was analyzed using inbuilt utilities of the GROMACS. The details of MD simulations have been described in our previous research [54,55].

## 4. Conclusions

Structure-based virtual screening was performed to identify some hits which may be promising inhibitors of CDK2. Using virtual screening and molecular docking studies, we found compound 101874157 which possesses a potent inhibitory potential for CDK2. The bioactivity of the selected compounds was predicted using theoretical approaches and the results showed that the identified compound 101874157 is chemically reactive and shows appreciable drug-like properties. MD simulations were performed on CDK2 in the free state and CDK2-101874157 complex to investigate their stability and dynamic behavior. Both CDK2 and CDK2-101874157 are quite stable throughout the simulation without any significant fluctuation. Overall, our findings provide enough evidence that the compound 101874157 might be a promising scaffold for CDK2 inhibition and can be further exploited as a lead molecule with required modifications to design potent and selective inhibitors of CDK2 to control cancer progression. In summary, the compound 101874157 promises a new gateway for the further development of anticancer therapeutics targeting CDK2.

## Figures and Tables

**Table 1:** ADME properties of compound 101874157.

Absorption		Distribution		Metabolism	Excretion		Carcinogenicity
WS *	HIA (%)	BBB Permeability	CNS Permeability	CYP Subs./Inh.	Clearance	Renal OCT2 Substrate	
Soluble	95.93	−0.83	−2.63	No	0.89	Yes	No

* WS is water solubility; HIA is human intestinal absorption: >90% is highly absorbed; BBB (blood brain barrier) penetration ability: <−1 are poorly distributed to the brain; CNS permeability: logPS <−3 are considered as unable to penetrate the CNS; CYP: CYP2D6 and CYP2C9; total clearance in log (mL/min/kg).

**Table 2:** Systematic and energetic parameters for both systems calculated after the simulation.

System	RMSD (nm)	RMSF (nm)	R*g* (nm)	SASA (nm^2^)	Kinetic Energy	Enthalpy	Volume (nm^3^)	Density (g/L)
CDK2	0.32	0.25	1.91	136.81	140,218	−723,310	571.86	1024.83
CDK2-101874157	0.31	0.23	1.94	139.49	140,064	−714,423	583.33	1004.62

**Table 3:** Percentage of residues participating in secondary structure formation of CDK2.

System	Percentage of Protein Secondary Structure							
	Structure *	Coil	β-sheet	β-bridge	Bend	Turn	α-Helix	Other ^#^
**CDK2**	58	28	14	1	12	11	32	2
**CDK2-101874157**	55	30	13	1	14	10	31	1

* Structure = α-helix + β-sheet + β-bridge + Turn; ^#^ Other = π-helix + 3_10_-Helix.

## Supplementary Materials

The following are available online, Figure S1: Interactions of CDK2 binding pocket with (A) docked compound 101874157, and reported co-crystalized inhibitors (B) Olomoucine, (C) Hymenialdisine, (D) SU9516 and (E) docked Bosutinib., Figure S2: Interactions of compound 101874157 with CDK2 before and after dynamic simulation., Table S1: Interacting residues of CDK2 PDB entries with their co-crystalized ligands. Number of bonds are mentioned in bracket., Table S2: Binding affinities of the selected PubChem compounds., Table S3: Physicochemical properties of the selected compounds with their consecutive values., Table S4: ADME properties of the selected compounds., Table S5: Chemical structures of the selected compounds along with their biological activity.
