# Supplementary material for: Identification of High-Affinity Inhibitors of Cyclin-Dependent Kinase 2 Towards Anticancer Therapy
Source: Molecules. 2019 Dec 15;24(24):4589. doi: 10.3390/molecules24244589 (PMC6943647; doi:10.3390/molecules24244589)
Supplement: Supplementary file 1 [file molecules-24-04589-s001.pdf]

Table S1: Interacting residues of CDK2 PDB entries with their co-crystalized ligands. Number of bonds are mentioned in bracket.

S. No.	PDB ID	Ligand ID	Participating residue in polar interaction
1.	1AQ1	STU	Glu81, Leu83, Asp86, Gln131 [2]
2.	1CKP	PVB	Leu83 [3]
3.	1DM2	HMD	Gly81 [2], Leu83 [2], Asp145 [2], Asn132
4.	1E1V	CMG	Glu81, Leu83
5.	1E1X	NW1	Lys33 [2], Glu81, Leu83
6.	1GIH	1PU	Lys33, Leu83 [3]
7.	1GII	1PU	Val83 [3], Asp145
8.	1GIJ	2PU	Val83 [3], Asp86, Asp145
9.	1GZ8	MBP	Glu172, Gly176 [2], Lys178, Tyr179
10.	1H0V	UN4	Lys33, Leu83 [2], Glu81, Asp86, Gln131 [2]
11.	1H0W	207	Glu81
12.	1FVT	106	Glu81, Leu83 [2], Asp86 [2]
13.	1G5S	I17	Leu83 [2], Asn132, Asp145
14.	1OIQ	HDU	Lys33, Asp145, Gly81, Leu83 [2]
15.	1OIR	HDY	Leu83 [2], Lys89
16.	1OIT	HDT	Lys33, Asp145, Leu83, Asp86 [3]
17.	1P2A	5BN	Glu81, Leu83 [3], Asn132, Asp145 [2]
18.	1PF8	SU9	Glu81, Leu83 [3]
19.	1PXI	CK1	Lys33, Glu81
20.	1PXJ	CK2	Lys33, Glu81, Leu83
21.	1PXK	CK3	Leu83 [4], His84, Asp145 [2]
22.	1PXL	CK4	Leu83 [3], Asp145 [2]
23.	1PXM	CK5	Leu83 [2], Asp86, Asp145
24.	1PXN	CK6	Leu83 [3], Lys33, Asp145
25.	1PXO	CK7	Ile10, Lys33, Asp145 [3], Leu83 [3], Asp86 [2]
26.	1PXP	CK8	Leu83 [2], Asp145
27.	2A0C	CK9	Leu83 [2], Gln131
28.	2BHE	BRY	Glu81, Leu83 [3]
29.	2BHH	RYU	Glu12, Glu81, Lys33, Leu83 [3], Asn132
30.	2DS1	1CD	Lys33, Val83 [3]
31.	2FVD	LIA	Glu81, Leu83, His84, Asp86, Lys89
32.	2R3F	SC8	Lys33, Leu83 [2]
33.	2R3G	SC9	Leu83, Asp86, Lys89
34.	2R3H	SCE	Leu83 [3]
35.	2R3I	SCF	Lys33 [2], Leu83 [4]
36.	2R3J	SCJ	Leu83 [2]
37.	2R3K	SCQ	Leu83 [2], Lys89
38.	2R3L	SCW	Leu83 [2]
39.	2R3M	SCX	Leu83 [2], Lys86 [2], Ile10
40.	2R3N	SCZ	Leu83 [2]
41.	2R3O	2SC	Leu83 [2]
42.	2R3P	3SC	Leu83 [4], Asp145 [2]
43.	2R31Q	5SC	Lys33, Leu83 [2]
44.	2R3R	6SC	Leu83 [3]
45.	2VTA	LZ1	Leu83 [2], Glu81
46.	2VTH	LZ2	Leu83, Glu81
47.	2VTI	LZ3	Leu83 [2], Glu81, Asp86, Lys89
48.	2VTJ	LZ4	Leu83 [2], Asp86 [2]
49.	2VTL	LZ5	Leu83 [3]
50.	2VTM	LZM	Leu83, Glu81
51.	2VTN	LZ7	Leu83 [2], Glu81
52.	2VTO	LZ8	Leu83 [2], Glu81
53.	2VTP	LZ9	Leu83 [2], Glu81
54.	2VTQ	LZA	Leu83 [2], Glu81, His84
55.	2VTR	LZB	Leu83 [2]

56.	2VTS	LZC	Lys33 [2], Leu83 [2], Asn132, Asp145
57.	2VTT	LZD	Leu83 [3], Glu81, Asp86
58.	2VU3	LZE	Tyr80, Arg180 [2]
59.	2W1H	L0F	Glu81 [2], Leu83 [2]
60.	3FZ1	B98	Lys33, Asp145 [2], Leu83, Asn132
61.	3LE6	2BZ	Glu81 [2], Leu83 [2], Asp86 [2]

Table S2: Binding affinities of the selected PubChem compounds.

S. No	PubChem ID	Affinity [Kcal/mol]
1.	117080005	-9.6
2.	117091375	-9.6
3.	117090929	-9.3
4.	10618917	-9.2
5.	117086854	-9.2
6.	118590042	-9.2
7.	71109332	-9.2
8.	10523324	-9.1
9.	44353621	-9.1
10.	67003239	-9.1
11.	118590044	-9.0
12.	6538928	-9.0
13.	67002909	-9.0
14.	10917098	-8.9
15.	117081323	-8.9
16.	59614560	-8.9
17.	67002677	-8.9
18.	117079230	-8.8
19.	117092467	-8.8
20.	17956382	-8.8
21.	25007915	-8.8
22.	6605150	-8.8
23.	6605230	-8.8
24.	67002002	-8.8
25.	67002045	-8.8
26.	854017	-8.8
27.	102366257	-8.7
28.	11048773	-8.7
29.	117081762	-8.7
30.	21922919	-8.7
31.	44315927	-8.7
32.	59068996	-8.7
33.	6538930	-8.7
34.	6605112	-8.7
35.	67002456	-8.7
36.	67002669	-8.7
37.	67002876	-8.7
38.	67003044	-8.7
39.	78074798	-8.7
40.	9933766	-8.7
41.	101874157	-8.6
42.	10228672	-8.6
43.	10806944	-8.6
44.	10961500	-8.6
45.	110154135	-8.6
46.	11198931	-8.6
47.	11416708	-8.6
48.	117080614	-8.6
49.	117081891	-8.6
50.	117082204	-8.6

Table S3: Physicochemical properties of the selected compounds with their consecutive values (MW = Molecular weight, LogP = lipophilicity, HBD = Hydrogen bond donor, HBA = Hydrogen bond scceptor, rBonds = Rotatable bonds, LV = Lipinski violation).

S. No.	Compound ID	MW	rBonds	HBA	HBD	iLogP	LV
1.	Olomoucine	298.34	6	4	3	2.62	0
2.	Hymenialdisine	324.13	0	3	4	0.92	0
3.	SU9516	241.25	2	3	2	1.31	0
4.	Bosutinib	530.45	9	7	1	4.68	1
5.	854017	309.37	4	4	1	3.00	0
6.	6605112	394.51	8	5	1	4.17	0
7.	6605150	380.49	6	5	2	3.40	0
8.	6605230	428.53	7	5	2	3.92	0
9.	9933766	456.58	8	4	3	4.26	0
10.	10228672	498.54	10	7	3	4.43	0
11.	10523324	372.47	7	3	2	3.73	0
12.	10618917	366.46	5	4	2	3.16	0
13.	10806944	338.41	5	4	2	2.19	0
14.	10917098	435.60	10	4	2	4.32	0
15.	11198931	351.45	5	4	2	3.38	0
16.	11416708	346.39	6	5	2	3.07	0

17.	17956382	467.30	6	6	1	3.69	0
18.	21922919	406.52	6	4	3	3.56	0
19.	25007915	374.44	6	4	3	3.24	0
20.	44315927	394.51	7	4	2	3.62	0
21.	44353621	406.52	6	4	2	4.12	0
22.	59068996	369.48	7	3	4	-0.81	0
23.	59614560	360.41	6	4	3	3.23	0
24.	67002002	431.53	8	4	4	3.45	0
25.	67002045	427.52	8	5	4	3.64	0
26.	67002456	409.53	7	4	4	3.57	0
27.	67002669	423.55	8	4	4	3.38	0
28.	67002677	417.51	8	4	4	3.32	0
29.	67002876	413.49	7	5	4	3.56	0
30.	67002909	421.47	8	5	4	3.33	0
31.	67003044	409.53	7	4	4	3.65	0
32.	67003239	441.54	8	5	4	3.79	0
33.	71109332	437.54	8	7	1	4.08	0
34.	78074798	430.55	10	4	3	4.28	0
35.	101874157	282.34	4	3	1	2.33	0
36.	102366257	385.46	8	4	2	3.79	0
37.	110154135	353.42	4	5	4	2.40	0
38.	117079230	392.50	5	4	2	3.58	0
39.	117080005	435.57	6	5	1	4.09	0
40.	117080614	435.57	6	5	1	4.22	0
41.	117081323	310.35	4	4	3	1.46	0
42.	117081762	331.37	5	4	2	2.98	0
43.	117081891	337.42	5	4	2	3.30	0
44.	117082204	346.39	5	4	3	2.79	0
45.	117086854	489.66	7	5	2	4.54	0
46.	117090929	435.57	8	5	2	4.22	0
47.	117091375	406.52	6	5	1	4.18	0
48.	117092467	353.42	7	5	3	2.07	0
49.	118590042	474.57	9	5	3	4.45	0
50.	118590044	474.57	9	5	3	4.84	0
51.	6538928	406.52	6	4	2	3.77	0
52.	6538930	414.50	7	4	2	3.54	0

Table S4: ADME properties of the selected compounds. Where- BBB is blood brain barrier penetration ability, HIA is human intestinal absorption, LogS is water solubility log mol/L, Log K_p is skin permeation (cm/s), TPSA is topological polar surface area, NC: Non-carcinogenic, and LV is lead-likeliness violation.

**S. No.**	**Comp. ID**	**BBB**	**HIA**	**LogK_p**	**CYP2C9 inhibitor**	**LogS**	**TPSA**	**Carcinogenicity**
1.	Olomoucine	No	High	-7.05	No	-2.95	87.89	NC
2.	Hymenialdisine	No	High	-8.39	No	-1.74	112.37	NC
3.	SU9516	Yes	High	-7.03	No	-2.04	67.01	NC
4.	Bosutinib	No	High	-5.72	Yes	-6.87	82.88	NC
5.	117081891	Yes	High	-6.19	No	-4.32	75.86	NC
6.	10806944	No	High	-6.81	No	-3.48	79.10	NC
7.	101874157	Yes	High	-6.30	No	-3.6	72.86	NC
8.	117081762	Yes	High	-6.18	Yes	-4.27	75.86	NC

Table S5: Chemical structures of the selected compounds along with their biological activity.

Compound	Chemical structure	Biological activity	References
101874157	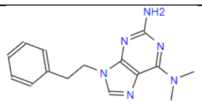	-	-
Olomoucine	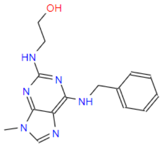	CDK2 inhibition	PMID:17139284, PMID:17016423, PMID:10592235, PMID:11752352
Hymenialdisine	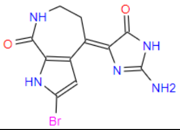	Stabilisation of CDK2	PMID:18077363
SU9516	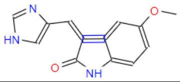	CDK2 inhibition	PMID:12234612, PMID:11507069
Bosutinib	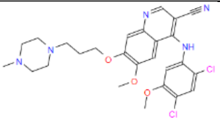	CDK2 inhibition	PMID:22037378

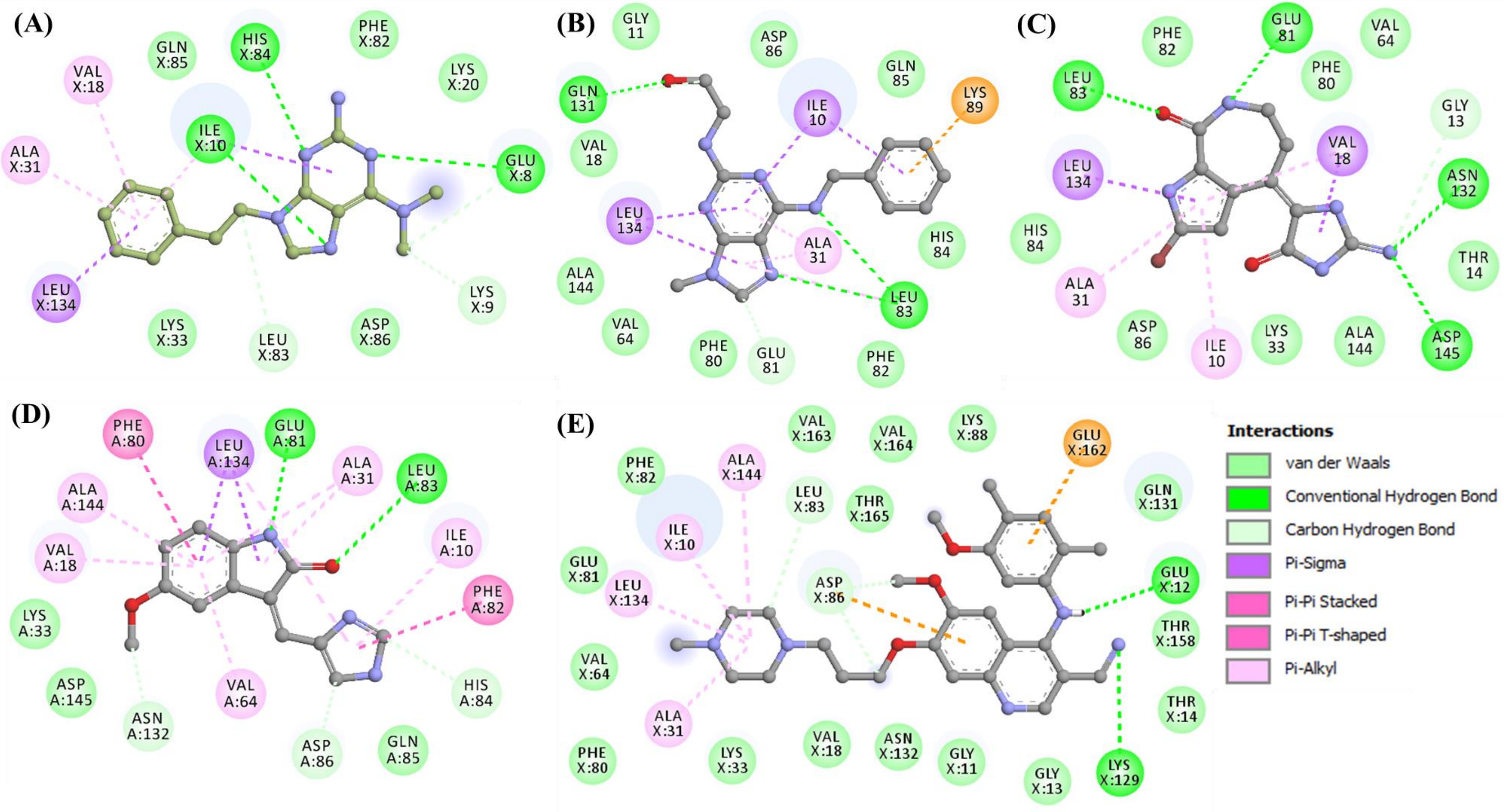

Figure S1: Interactions of CDK2 binding pocket with (A) docked compound 101874157, and reported co-crystalized inhibitors (B) Olomoucine, (C) Hymenialdisine, (D) SU9516 and (E) docked Bosutinib.

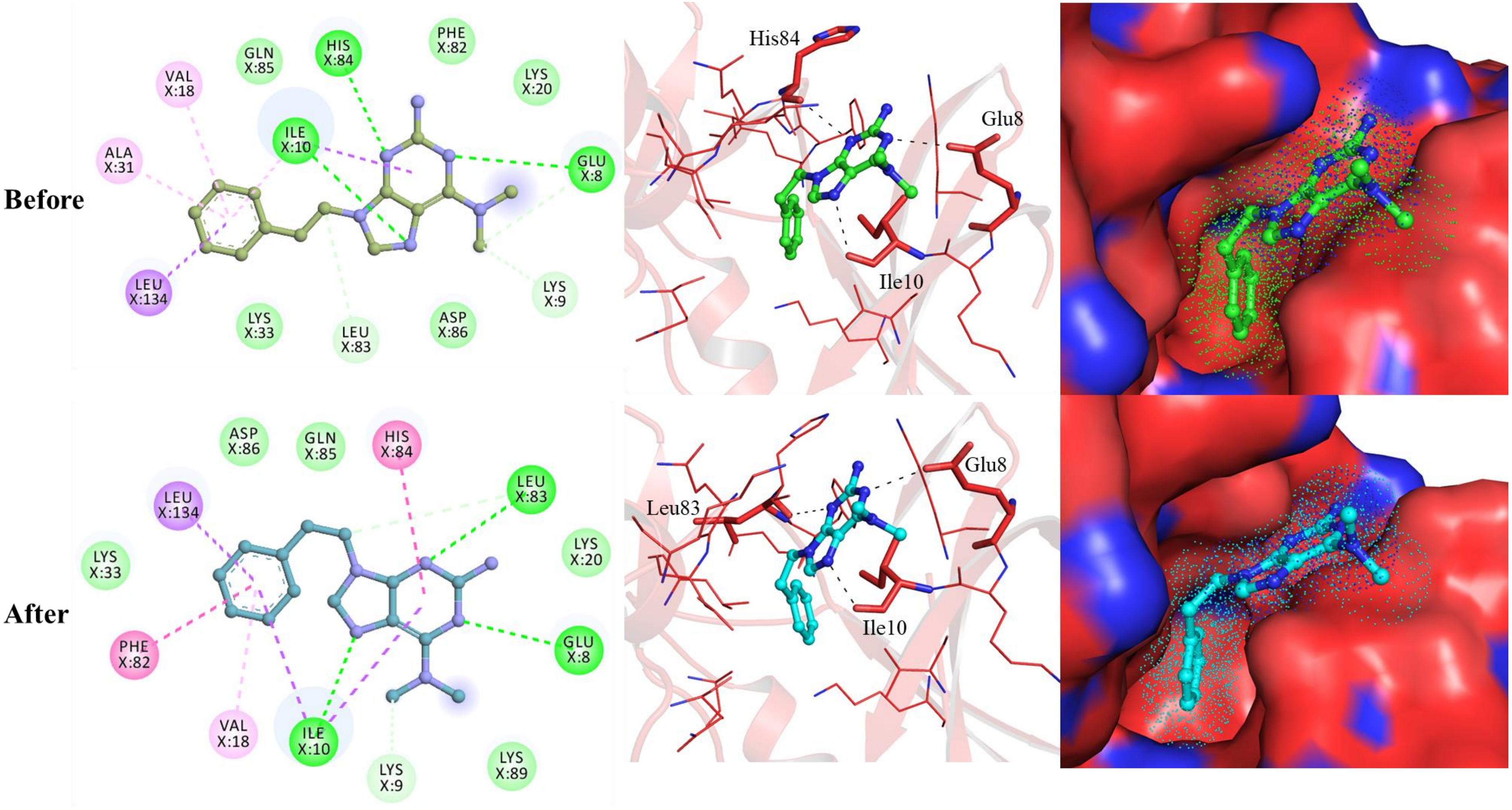

Figure S2: Interactions of compound 101874157 with CDK2 before and after dynamic simulation.
